# Aesthetic preferences for prototypical movements in human actions

**DOI:** 10.1186/s41235-023-00510-0

**Published:** 2023-08-17

**Authors:** Yi-Chia Chen, Frank Pollick, Hongjing Lu

**Affiliations:** 1 Department of Psychology, University of California, Los Angeles, USA; 2 School of Psychology and Neuroscience, University of Glasgow, Glasgow, UK; 3 Department of Statistics, University of California, Los Angeles, USA

**Keywords:** Category, Prototype, Aesthetics, Biological motion, Emotion

## Abstract

A commonplace sight is seeing other people walk. Our visual system specializes in processing such actions. Notably, we are not only quick to recognize actions, but also quick to judge how elegantly (or not) people walk. What movements appear appealing, and why do we have such aesthetic experiences? Do aesthetic preferences for body movements arise simply from perceiving others’ positive emotions? To answer these questions, we showed observers different point-light walkers who expressed neutral, happy, angry, or sad emotions through their movements and measured the observers’ impressions of aesthetic appeal, emotion positivity, and naturalness of these movements. Three experiments were conducted. People showed consensus in aesthetic impressions even after controlling for emotion positivity, finding prototypical walks more aesthetically pleasing than atypical walks. This aesthetic prototype effect could be accounted for by a computational model in which walking actions are treated as a single category (as opposed to multiple emotion categories). The aesthetic impressions were affected both directly by the objective prototypicality of the movements, and indirectly through the mediation of perceived naturalness. These findings extend the boundary of category learning, and hint at possible functions for action aesthetics.

**Supplementary Information:**

The online version contains supplementary material available at 10.1186/s41235-023-00510-0.

## Introduction

Living in a world full of objects and events, there is one kind of stimuli that captivates most of us: other people. Our visual systems specialize in processing sights related to other people, including their eyes (Emery, 2000), faces (Kanwisher & Yovel, 2006), bodies (Peelen & Downing, 2007), and even human-designed objects (Lopez-Brau et al., 2021). Upon seeing other people, in addition to recognizing identity, emotion, and gender, a wealth of other subjective impressions also arise naturally. Most notably, we are very quick to notice how attractive others appear—a mere glance gives rise to an aesthetic experience (Willis & Todorov, 2006). These aesthetic impressions are not only quick, but also impact important aspects of our lives (e.g., dating and hiring decisions, Marlowe et al., 1996).

How do these aesthetic impressions of other people arise? Extensive research has uncovered various perceptual factors that determine facial and body attractiveness, including shape averageness, symmetry, and sexual dysmorphism (Thornhill & Gangestad, 1999; Fan et al., 2004, 2005). However, if the aim is to fully understand our aesthetic impressions of other people, many researchers have noted some missing pieces (Fink et al., 2015; Johnson & Tassinary, 2007; Morrison et al., 2018): Most of this past research has used static images or illustrations as stimuli. However, we do not often see completely static people in our lives, especially not so in the evolutionary past. People move, and these movements often signal critical social information, such as emotional states (Pollick et al., 2001), goals (Csibra et al., 1999), and social intentions (Barrett et al., 2005; Colombatto et al., 2020). In this spirit, we aimed to explore how body movements give rise to aesthetic experiences.

Understanding how people’s dynamic “looks” give rise to aesthetic experiences is important for solving several real-world problems: First, to curb the (sometimes undesirable) impact of appearances on our personal and professional lives, it is critical to first understand what kind of aesthetic experiences can arise upon seeing a person. This study aimed to fill the gap between the vast scientific explorations on static stimuli and dynamic real-world experiences. Second, knowledge of action aesthetics will aid the design and development of the virtual world, such as those in animations, avatars in virtual reality, and robotics. Third, being able to evaluate human movements plays critical roles in medical diagnosis and rehabilitation (e.g., Sparrow et al., 2002). We aimed to develop necessary computational models that can provide quantitative measures in these clinical needs.

Beside the practical needs of understanding aesthetics in movements, the present study also contributes theoretically to three fields: aesthetic perception, categorical processing, and biological motion perception. First, we assessed the extent to which aesthetic experiences exhibit systematic regularities from body movements. We used a type of dynamic stimuli that is frequently experienced—human walking. Also, to further understand what kind of perceptual processing gives rise to these aesthetic experiences, we included walking actions indicative of different emotion states. This allowed us to tease apart effects of motion perception from social perception of emotion. The choice to use everyday stimuli like human walking was intentional, as we aimed to research a different aesthetic experience from what previous research has focused on. Specifically, for the aesthetics of movement, there exists an interdisciplinary field of research on the aesthetics of dance (for a review, see Christensen et al., 2013). This aesthetic research has examined artistic movements explicitly designed to communicate with and elicit various emotional and aesthetic experiences in an attentive and interested audience (for a discussion, see Orlandi et al., 2020). Our goal, however, was to look at the spontaneous aesthetic experiences that arise from seeing everyday stimuli, which naturally carry biological and social information without artistic or communicative intentions (for a discussion on the distinction of art and aesthetics research, see Palmer et al., 2013).

Second, we used an aesthetic phenomenon—the preferences for category prototypes—as a lens to study how people organize representations of actions into different categories: Prototype preferences underly all sorts of visual categories including human faces (Galton, 1878; Langlois & Roggman, 1990), artificial or realistic biological organisms (Halberstadt & Rhodes, 2003; Younger, 1990), man-made objects (Landwehr et al., 2011; Whitfield & Slatter, 1979), abstract shapes (Solso & Raynis, 1979), and dot patterns (Posner & Keele, 1968). However, most of these past explorations focused on static stimuli (except for a few pioneering studies, Ackermann & Adams, 2004; Sparrow et al., 2002). Thus, we asked: do dynamic events like movements in walking actions also lead to prototype representations and preferences? With this approach, we also asked what the potential function of categorical processing for actions may serve. Do the categorical representations serve to recognize different kinds of actions, or could it be further involved in perceiving the social characteristics these actions may imply? To probe this question, we further assessed whether the potential prototype effects reflect a unified category of human walking actions, or multiple subcategories conditional on different emotion states that often associate with different underlying social intentions.

Third, the choice to investigate a possible aesthetic prototype effect also represents a new approach to understand biological motion processing. Explanations of aesthetic experiences from actions proposed in past studies have been based almost exclusively on domain-specific processes for human movements. For example, men’s dance movements are linked to attractiveness because dances are used as demonstrations of physical abilities (Hugill et al., 2009; McCarty et al., 2013), and women’s gait patterns are linked to attractiveness as they signal their fertile period around ovulation (Fink et al., 2012). Specialized processing of biological motion has also been proposed to explain a preference for consistency between body shape and movements (Klüver et al., 2016). These studies suggested that the specialized perceptual processes for biological motions underly specific aesthetic preferences that serve unique functions. Here, we asked a new question: Beyond specialized aesthetic effects, can domain-general aesthetic effects such as the prototype effect be observed in human actions? Answering this question helps situate the role of general perceptual processing in seeing biological motion.

We addressed these real-world needs and theoretical questions by conducting three behavioral experiments to measure observers’ aesthetic impressions of walking actions from different actors expressing different emotions, and by constructing computational models based on the prototypicality of human walking to account for observed aesthetic experiences. To isolate the effect of action dynamics, we used point-light displays from a motion capture dataset (Ma et al., 2006) to remove the influence of body shape appearance on the walkers. In the first experiment, we asked how much consensus and systematicity there are in aesthetic experiences from seeing other people walk. In the second experiment, we explored how emotion recognition and human form influences these aesthetic experiences. In the third experiment, we examined how action prototypicality and aesthetic impressions are linked causally.

## Method

### Participants

For each of the three experiments, 50 naive observers (Experiment 1: 34 females and 16 males; Experiment 2: 33 females and 17 males; Experiment 3: 40 females and 10 males; all with normal or corrected-to-normal vision) from the University of California, Los Angeles (UCLA) community completed an individual 30-min experimental session online in exchange for course credit. A total of 40 additional observers participated (15 in Experiment 1; 15 in Experiment 2; 10 in Experiment 3) but were removed based on predetermined criteria (see details in the Observer exclusions section below). The sample size was predetermined arbitrarily, preregistered, and fixed throughout all experiments. The study was approved by the UCLA Institutional Review Board.

### Stimuli

Because the stimuli were rendered on observers’ own web browsers, viewing distance, screen size, and display resolutions could vary depending on computer monitors used by observers; hence, we report visual stimulus dimensions using pixel (px) values.

#### Intact walker videos for Experiment 1 and 3

**Fig. 1:**
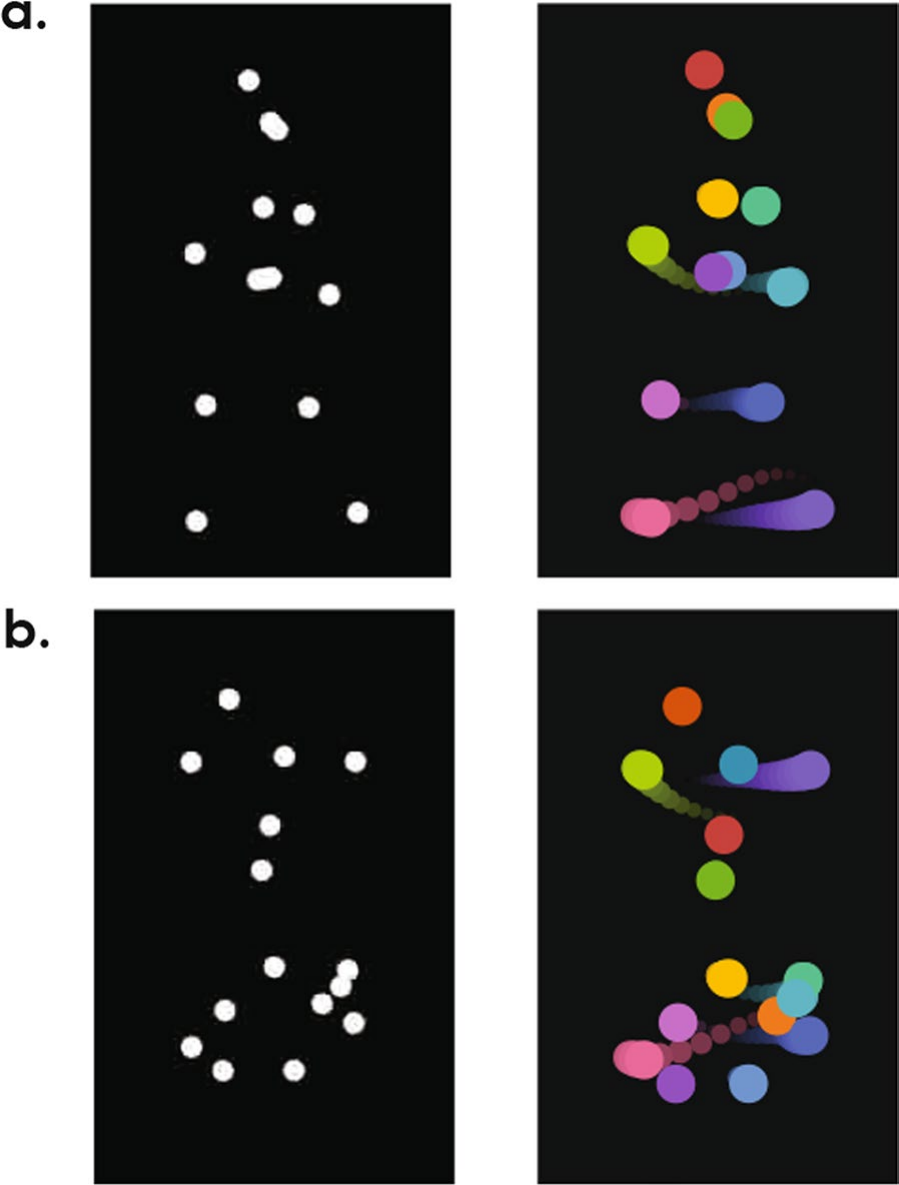
**a** The image on the left is a sample frame from one of the intact walker videos used in Experiment 1 and 3. The image on the right depicts a few frames of movements after removing the global body displacements in the video. **b** A sample frame of the same walker from (**a**) in a scrambled video used in Experiment 2 and its corresponding movement depiction

From the motion capture dataset (Ma et al., 2006), we created 80 point-light displays from 20 unique walkers (10 female, 10 male) expressing four different emotions (happy, neutral, angry, sad) while walking back and forth between left and right. For each emotion, actors read a script that depicted different emotional scenarios for them to express through their movements. For example, the script for performing the happy walking action was “It’s Friday evening and you feel great, because earlier you handed in your final year project. Your supervisor was very pleased, he complimented you on it and hinted that you’re going to get excellent marks for it. You just talked you your flatmate who suggested you go out to celebrate and now you are just waiting for him to finish getting ready. As you are getting more excited, you start pacing around the room, this is going to be such a good night and you can’t believe that you are almost finished with your degree. You almost want to start skipping with joy!”. The same procedure also applied to the neutral walking action, with the script read: “It is a sunny Saturday morning and you are in your flat, the sun is streaming in through the windows and you are relaxed and well rested. You are waiting for your flat mate since the two of you are going out shortly. While waiting you start pacing, more from habit than anything else.” All actors were given the same scripts to read before performing actions. Details of creating the motion capture dataset and instructions were included in the paper by Ma and colleagues (2006). For the present study, each walking video was created by using the 5-s excerpt after 8.3 s (500 frames in 60 Hz sampling rate) in the motion-capture film. We then down-sampled the video from 60 to 30 Hz, which is a more typical frame rate for videos displayed online. Fifteen joints were used to create each point-light display (650px × 350px), and each joint was depicted as a white dot (12px in diameter) on a uniform black background (Fig. 1a). The videos were then mirrored to create the 80 mirrored videos.

#### Spatially scrambled creature videos for Experiment 2

To isolate factors related to local joint motion from the holistic processing and explicit recognition of emotion expressions, we created spatially scrambled videos so that the configuration of body shapes no longer resembled a human walker. This was achieved by randomizing initial positions of each of the 15 joints in each walker while keeping the trajectory patterns of the joints intact (Fig. 1b; Cutting, 1981). We selected random initial x and y positions separately. For y positions, we first found the full y movement ranges throughout the 5-s video for each walker and each of their 15 joints. We then randomly selected a new y position for each joint, with the constraint that the full movement range of the joint stay inside the full movement range for the walker. For x positions, we calculated the joints’ relative x positions from the center of each walker (mean x position of all joints) in each frame of the 5-s videos. This way we temporarily removed the global horizontal motion of the walkers for the randomization process. We then performed the same position selection used for y positions. Finally, we added the horizontal movements back to all joints in each frame. The videos were then mirrored to create the 80 mirrored videos. These spatially scrambled point-light displays were referred to as “creatures” in the experiment.

### Experiment procedure

Observers were directed to a website where stimulus presentation and data collection were controlled via custom software written in HTML, CSS, JavaScript, JQuery, and PHP. Observers were not allowed to participate using phones or tablets. After completing a CAPTCHA task (using the hCaptcha service: https://www.hcaptcha.com/), they were asked to maximize their window size, informed about their task, and quizzed about their understanding of the instructions, and then, they provided their consent. During the instructions, they were shown 4 videos from different walkers and different emotional expressions, to help them gauge the range of experiences they would have. They then performed one practice trial to get familiar with the rating scale.

In the first part of Experiment 1, observers were asked to rate “how visually pleasing you find each walking style to be”, and “In other words, how good/beautiful do you think the walking style looks/movements look”. The same goes for the first part of the Experiment 2, except the phrase “walking style” was replaced with “creature's movement”. Observers rated each video on a 6-point likert scale with labels (certainly pleasing, probably pleasing, guess pleasing, guess not pleasing, probably not pleasing, and certainly not pleasing). For Experiment 3, instead of aesthetic ratings, the observers provided ratings to indicate their subjective impression of each walking style’s prototypicality. To avoid jargon, we did not use the word prototypicality directly in the instructions, but asked the observers to rate the walking styles’ naturalness (“……how natural you find each walking style to be. In other words, how common/usual do you think the walking style looks”). Again, they used a 6-point scale with naturalness labels (certainly natural, probably natural, guess natural, guess not natural, probably not natural, and certainly not natural).

In the second part of all three Experiments, the observers rated the emotion positivity of the walkers (Experiment 1 and 3) or the creatures (Experiment 2) in the videos (“……how positive you find each walker/creature’s emotion to be. In other words, how positive of a mood do you think the walker/creature is in”), using a 6-point scale with emotion positivity labels (certainly positive, probably positive, guess positive, guess not positive, probably not positive, and certainly not positive).

The videos displayed intact walkers in Experiment 1 and 3, and spatially scrambled walkers in Experiment 2. Each of the 80 videos was displayed once in each block in different random orders. Whether the original or the mirrored version were shown was randomly decided for each video and each observer but kept the same across the two rating tasks.

At the end of the experiment, observers answered a series of debriefing questions to ensure they had completed the experiment without any issues.

### Observer exclusions

In addition to the 150 observers whose data were analyzed, 40 observers (15 in Experiment 1; 15 in Experiment 2; 10 in Experiment 3) participated and were excluded based on criteria decided before data collection began, with some observers triggering more than one criterion. For Experiment 1, five observers reported that they did not follow the instructions or did not take the experiment seriously; one observer failed the instruction quiz more than once; one observer spent less than 0.5 s on at least one page of the instructions; one observer had a browser viewport smaller than 800px × 600px; one observer had at least one trial with the video not fully in view during the rating task; one observer gave the same rating to more than 15 consecutive trials; six observers hid the experiment browser tab more than three times during the trials; and four observers took too long to complete the experiment (two SDs longer from the mean duration from all observers in the same experiment before exclusions).

For Experiment 2, seven observers reported that they did not follow the instructions or did not take the experiment seriously; one observer spent less than 0.5 s on at least one page of the instructions; three observers had a browser viewport smaller than 800px × 600px; one observer had more than four trials with response times longer than two minutes in at least one block; two observers hid the experiment browser tab more than three times during the trials; one observer provided a non-sensical answer to one of the debriefing questions; and three observers took too long to complete the experiment.

For Experiment 3, one observer reported technical issues, three observers reported that they did not understand the instructions or did not take the experiment seriously; one observer spent less than 0.5 s on at least one page of the instructions; one observer had at least one trial with the video not fully in view during the rating task; three observers hid the experiment browser tab more than three times during the trials; and two observers took too long to complete the experiment.

### Prototypicality model

#### Trajectory preprocessing

Each video was preprocessed separately following these steps. (a) We first subtracted the head’s x position in each frame from all joints’ x positions to remove the global horizontal movement. (b) The mean x and y positions for each joint across all the frames were calculated and subtracted from the joint’s x and y positions in each frame. This way, all joints’ trajectories ended up centering at coordinate (0, 0). (c) The video was segmented manually into multiple action clips based on the type of movements the walker was performing—walking from left to right, turning around on the right, walking from right to left, or turning around on the left. The number of action clips differed between videos and ranged from 1 to 5 clips.

#### Dynamic time warping

Since the same type of movements in different action clips was performed in each walker’s own walking speed and rhythm. Comparing different clips required a method to map the corresponding frames in the gait cycle across walkers. For example, the frame where Walker A raised their right foot in Clip A should be compared with the frame where Walker B raised their right foot at a different time point in Clip B. We used dynamic time warping algorithms (DTW) to measure dissimilarity between walking sequences. DTW was performed by “warping” the temporal sequences of coordinates nonlinearly in time to find the optimal (i.e., least dissimilar) correspondence between two sequences. We use a simple example to illustrate this algorithm: Considering computing dissimilarity between two sequences of 2D coordinates—A sequence: [(0,0), (0,0), (1,2), (3,3)] and B sequence: [(0,0), (2,2), (3,3), (3,3), (3,4)], the algorithm would correspond A1 (i.e., the coordinates in the first frame of A sequence) and A2 (the second frame of A sequence) both to B1 (the first frame of B sequence), and A3 to B2, and finally A4 to B3, B4, and B5. With this correspondence mapping over time, the algorithm can minimize the total dissimilarity, calculated by the distance between matched coordinates (0 + 0 + 1 + 0 + 0 + 1 = 2). This procedure allowed us to map corresponding frames between two clips in an automatic and data-driven manner.

#### Prototypicality scores

To model prototypicality, we performed pairwise comparison between all 80 videos. For each action clip in each video, we applied DTW algorithm multiple times to find the best matching sequence in all other 79 videos following these steps: (a) We measured the clip length in frame number and dropped any action clip that had less than six frames (200ms) as they contained too little information for meaningful mapping. (b) For each of the rest of the action clips, we found the best matching sequences within the full length of each of the other 79 videos. We did so by defining the max and min clip length for the possible matching sequence as ± 12 frames from its own clip length, with the constraint that the length should be no longer than 150 frames (the full video) and no shorter than seven frames. (c) We looped through all allowed clip lengths for the possible matching sequences. For each clip length, there will be multiple possible matching sequences in each of the other 79 videos. For example, a clip length of 5 frames includes sequences like Frame 1–5, Frame 2–6, Frame 3–7, and so on, from another video. We performed DTW between the action clip and each of these possible matching sequences to find the one that had the smallest dissimilarity across clip length. Thus, we found the best corresponding sequence and its dissimilarity to the action clip for each of the other 79 videos. Note that the DTW was performed considering all 15 joints’ coordinates at the same time.

The total dissimilarity between two videos was then calculated by summing over the dissimilarity of each action clip in one video and their best corresponding sequences in the other video. This dissimilarity score was then normalized to the standard video length of 150 frames (since a few action clips might be dropped from finding a corresponding sequence because of its short clip length). Note that this process yielded asymmetrical dissimilarity scores when we find the matching sequences from Video A to Video B, and Video B to Video A. We simply took the smaller value to represent two videos’ dissimilarity. A video’s prototypicality score was calculated by one divided by its average dissimilarity score with all other videos in a category (i.e., the other 79 videos for the one-category model). Hence, higher prototypicality score of a video indicates that the video is more representative of a category as it shows higher average similarity to all exemplars in this category.

### Mediation analysis

To understand the causal path between objective prototypicality and aesthetic impression, we conducted a mediation analysis to separate direct effect and indirect effect through subjective prototypicality. Beside the standard Sobel test, we performed the “permutation confidence interval for ab” method (Taylor & MacKinnon, 2012) to address the caveats of the Sobel test. Using both the *z*-scores of the ratings and the residuals after regressing out emotion positivity ratings, we followed these steps: (a) We first calculated the predicted aesthetic rating for each video according to a linear regression model including both modeled prototypicality and naturalness rating as predictors. (b) The aesthetic ratings’ residuals from these predicted values were calculated. (c) We calculated the predicted naturalness rating for each video according to a linear regression model including only modeled prototypicality as the predictor. (d) The naturalness ratings’ residuals from these predicted values were calculated. (e) We iterated the following steps (f-j) for 10,000 times. (f) The residuals calculated from step b were permutated and led to a permutated set of aesthetic ratings. (g) The same permutation was applied to naturalness residuals and led to a permutated set of naturalness ratings. (h) Using a linear regression model with model prototypicality as the predictor to predict permutated naturalness ratings, we found the permutated slope for prototypicality (denoted by a*). (i) Using a linear regression model with both modeled prototypicality and the original naturalness rating as predictors to predict permutated aesthetic ratings, we found the permutated slope for naturalness (denoted by b*). (j) We then multiplied a* and b* to get the permutated indirect effect. (k) After 10,000 iterations, we got a distribution of a* x b* values and found the confidence interval based on the 250^th^ and the 9751^st^ values after sorting.

### Transparency and openness

In the above sections, we reported all data exclusions, all manipulations, and all measures. Experiment 1’s design and analysis plan were preregistered and can be viewed here: https://aspredicted.org/KJD_33W. All materials, code, and data can be downloaded here: 10.17605/OSF.IO/87G3E. A demonstration of the experiments can be viewed online here: https://yi-chia-chen.github.io/walker-prototype-demo-expt/.

## Results

### Behavioral findings: emotion expressions influence aesthetic impressions

We first addressed two questions regarding the patterns in the behavioral measures: Is there a systematic consensus in people’s aesthetic impressions evoked from point-light walkers? How do the emotion expressions relate to aesthetic experiences?

#### Systematic consensus in action aesthetics

To gauge the consensus on aesthetic impressions, we used data from Experiment 1 and calculated each observer’s “aesthetic taste typicality” (Chen et al., 2022a) by correlating their rating *z*-scores for each video to the average *z*-scores of the other 49 observers for each video. This measure revealed how similar an observer’s aesthetic taste was compared to an average taste from all other observers—hence, how typical one’s aesthetic taste was. All observers showed positive taste typicality, except for one observer who showed a weak negative taste typicality (Experiment 1: *M* = 0.536, SD = 0.176, Range = [− 0.099, 0.788]; Experiment 2: *M* = 0.272, SD = 0.139, Range = [− 0.010, 0.532]). This pattern indicates substantial consensus across observers and systematic variations in aesthetic impressions across different walks.

#### Emotion positivity correlated with positive aesthetic impressions

**Fig. 2:**
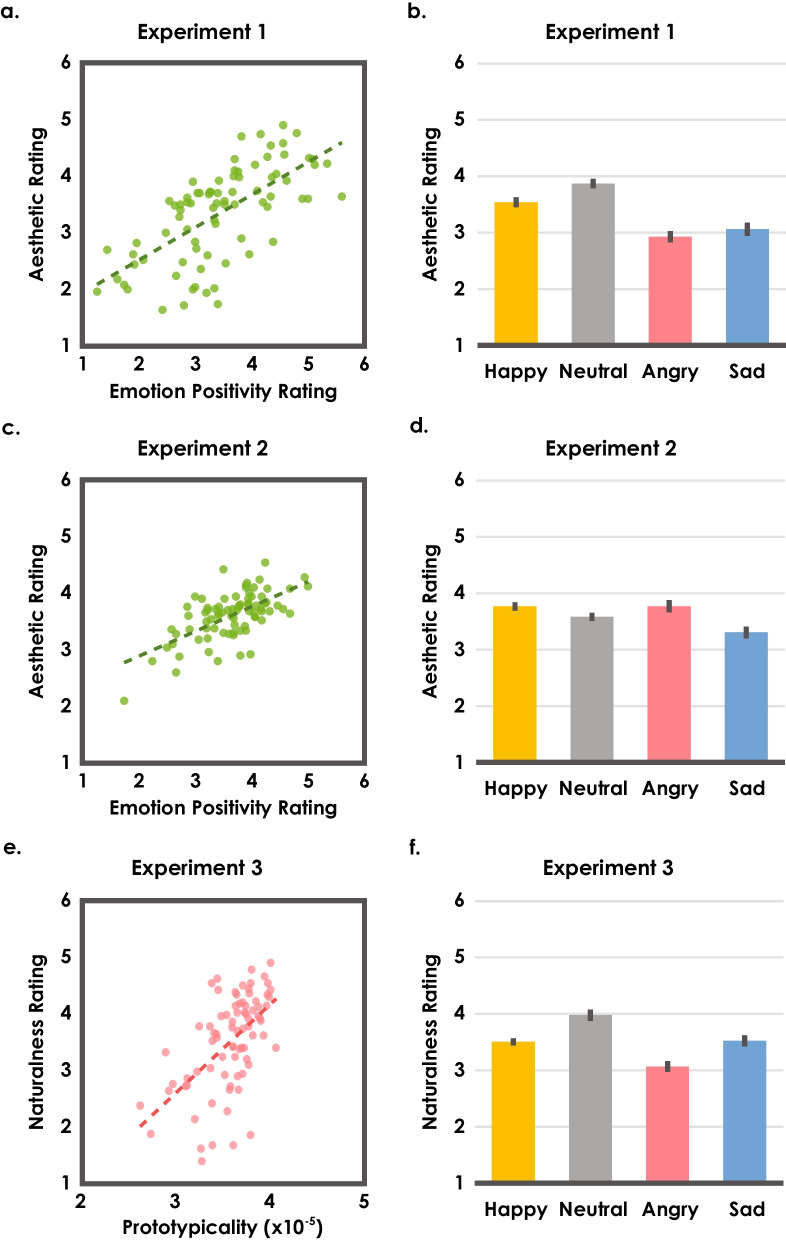
**a** Each intact walker video’s mean aesthetic rating from Experiment 1 plotted against its emotion positivity rating. **b** Mean aesthetic ratings of videos in four emotion categories from Experiment 1. All error bars are within-subject 95% confidence intervals (computed after subtracting individual overall means from the individual’s means in four categories). **c** Each scrambled creature video’s mean aesthetic rating from Experiment 2 plotted against its emotion positivity rating. **d** Mean aesthetic ratings of scrambled creatures in four emotion categories from Experiment 2. **e** Each intact walker video’s mean naturalness rating from Experiment 3 plotted against its modeled prototypicality. **f** Mean naturalness ratings of videos in four emotion categories from Experiment 3

For both Experiment 1 (intact walkers) and Experiment 2 (scrambled creatures), we depict each video’s mean aesthetic and emotion positivity ratings in Fig. 2a, c. There is a clear relationship between aesthetic ratings and emotion positivity ratings: The more emotionally positive a walk appeared, the more aesthetically pleasing it looked. This pattern was confirmed by a significant positive by-video correlation calculated with group averages of *z*-scores (Intact walkers: *r*(78) = 0.662, *p* < 0.001; Scrambled creatures: *r*(78) = 0.627, *p* < 0.001; all tests reported were two-tailed tests), and further supported by a one-sample t-test of comparing correlations between the two ratings at the individual level to zero (Intact walkers: *M* = 0.321, SD = 0.186, Range = [− 0.206, 0.713]), *t*(49) = 12.18, *p* < 0.001, Cohen’s* d* = 1.72; with 48/50 observers showing positive correlations, *p* < 0.001; Scrambled creatures: *M* = 0.186, SD = 0.174, Range = [− 0.168, 0.729]), *t*(49) = 7.59, *p* < 0.001, Cohen’s *d* = 1.07; with 42/50 observers showing positive correlations, *p* < 0.001).

#### Emotion categories revealed sophisticated effects

If the effect of emotion positivity was unidimensional (differing only with respect to how positive the expressions appeared), the relationship between aesthetic and emotion positivity ratings described above would predict that happy walkers/creatures would yield most positive aesthetic impressions compared to neutral, angry, and sad walkers/creatures. However, we found a different patterns of results: In Experiment 1 with intact walkers, when different emotional categories were examined (Fig. 2b), the neutral walkers were rated the most aesthetically pleasing, even higher than the happy walkers (*M*_happy_ = 3.5 (0.6), *M*_neutral_ = 3.9 (0.5), *M*_angry_ = 2.9 (0.6), *M*_sad_ = 3.1 (0.6); and showed a significant main effect of emotion category on aesthetic ratings (*F*(3, 147) = 62.0, *p* < 0.001, *η*^*2*^_*p*_ = 0.559; all post hoc comparison with neutral walkers: ts > 4.4, ps < 0.001 after Bonferroni correction; see Additional file 1 for details). Moreover, this finding was not due to misclassification of the walkers’ emotion expressions, as the happy walkers were still rated the most emotionally positive (*M*_happy_ = 4.0 (0.4), *M*_neutral_ = 3.7 (0.4), *M*_angry_ = 3.4 (0.5), *M*_sad_ = 2.6 (0.4); one-way ANOVA main effect with *z*-scores: *F*(3, 147) = 185, *p* < 0.001, *η*^*2*^_*p*_ = 0.791; all post hoc comparison with happy walkers: ts > 5.9, ps < 0.001 after Bonferroni correction). Furthermore, the effect of neutral walkers having higher aesthetic ratings than other emotion categories of walkers remained to be significant after regressing out the emotion positivity ratings from the aesthetic ratings (one-way ANOVA main effect with aesthetic residuals: *F*(3, 147) = 41.0, *p* < 0.001, *η*^*2*^_*p*_ = 0.455; all post hoc comparison with neutral walkers: ts > 5.8, ps < 0.001 after Bonferroni correction). In Experiment 2, when body configuration was removed by spatial scrambling, this effect was not observed (Fig. 2d): Observers found happy and angry creatures more aesthetically pleasing than neutral creatures, and sad creatures the least aesthetically pleasing (*M*_happy_ = 3.8 (0.4), *M*_neutral_ = 3.6 (0.4), *M*_angry_ = 3.8 (0.6), *M*_sad_ = 3.3 (0.5); main effect with z-scores: *F*(3, 147) = 18.7, *p* < 0.001, *η*^*2*^_*p*_ = 0.276).^1^ This finding suggested that the pattern of results observed in intact walkers depends on explicitly recognizing human actions, as a different result pattern emerged when the moving entity no longer appeared to be a human being.

Why do we aesthetically prefer neutral walkers? If categorical representations based on prototypes exist for human walkers, it is possible that neutral walkers appeared the most prototypical, and thus, the preference is a result of an aesthetic prototype effect. To test this idea, we used a computational model to quantify objective prototypicality in walking stimuli, and in Experiment 3 we measured observers’ subjective prototypicality.

### Computational modeling: an aesthetic prototype effect in human walks

**Fig. 3:**
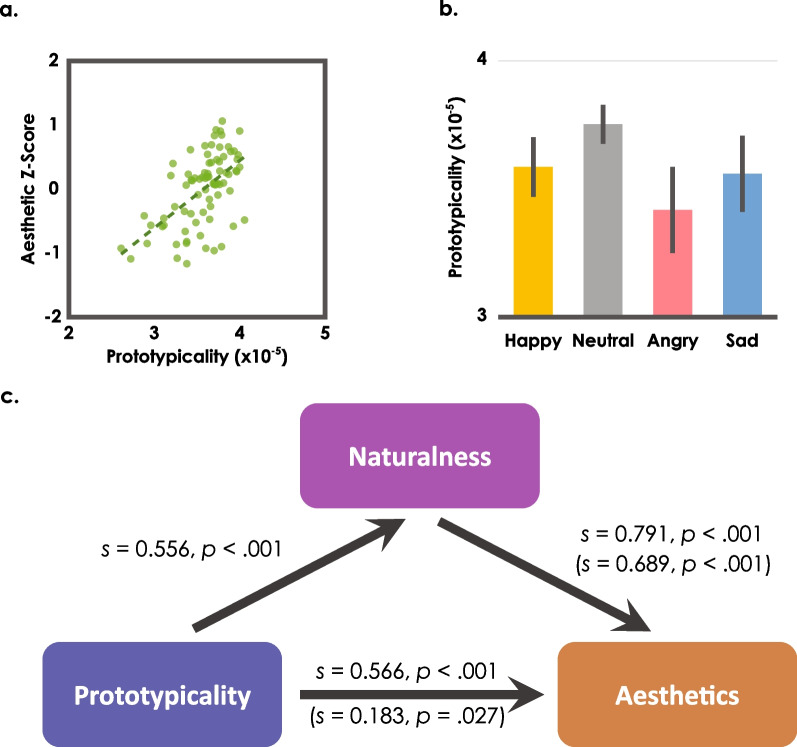
**a** Each intact walker video’s mean aesthetic rating from Experiment 1 plotted against its single-category objective prototypicality. **b** Mean single-category objective prototypicalities from intact walker videos in four emotion categories. **c** The modeled prototypicality’s effect on the aesthetic *z*-scores was partially mediated by the naturalness *z*-scores. Here, *s* represents the slope. The results of regressions with the indirect effect of naturalness removed are reported in parentheses

We first constructed a single category model: Using the dynamic time warping algorithm (DTW) to compute the similarity of joint movements from two actions (Gavrila & Davis, 1995), we calculated pairwise similarity across walking videos and computed average similarity for each walker (to the other 79 walks) as an index of objective prototypicality. Body movements closer to the prototypical walking sequence would show greater similarity to the other walks, resulting in greater prototypicality index values. We then examined the relation between the objective prototypicality index for each of the walks and the aesthetic ratings from Experiment 1 for intact point-light walkers: We found that the more prototypical a walk was, the more aesthetically pleasing it appeared both before and after regressing out the emotion positivity ratings (Fig. 3a; aesthetic *z*-scores average: *r*(78) = 0.566, *p* < 0.001, aesthetic residuals average: semipartial correlation *r*(78) = 0.546, *p* < 0.001). Critically, the objective prototypicality index revealed that the neutral walkers indeed were the most prototypical (Fig. 3b), followed by happy, sad, and angry walkers (one-way ANOVA main effect: *F*(3, 57) = 6.91, *p* < 0.001, all post hoc comparison with neutral walkers: ts > 2.9, ps < 0.05 after Bonferroni correction). Thus, the computational model provides an account for the aesthetic prototype effect, supporting the hypothesis that dynamic events involve representations of a category.

Walking stimuli in our study might be represented as exemplars of a single category (walking), or as exemplars of multiple categories depending on the different emotions the walkers were expressing (e.g., happy walking, sad walking). Would a model with more refined emotional categories more accurately predict human aesthetic judgments relative to the parsimonious model with a single category of walking? To address this question, we tested the emotion category model: For each walk, instead of computing the overall average similarity to the other 79 walks, we computed average similarity only within the same expression (to the other 19 walks).^2^ We then examined the relation between the emotion category objective prototypicality index for each of the walks and the aesthetic ratings from Experiment 1 in the same way as for the single category model: we again found that the more prototypical a walk was, the more aesthetically pleasing it appeared (*r*(78) = 0.382, *p* < 0.001). However, this correlation from the emotion category model was weaker than that from the single category model (emotion category model: *r*(78) = 0.382, single category model: *r*(78) = 0.566; comparison: *p* = 0.012; all comparisons between correlations were conducted with cocor, Diedenhofen & Musch, 2015, based on Pearson & Filon, 1898). After regressing out the emotion positivity ratings from the aesthetic ratings, the single category model still out-performed the emotion category model (semipartial correlation *r*(78) = 0.440, *p* < 0.001) numerically (single category model: semipartial correlation *r*(78) = 0. 546; comparison: *p* = 0.138). Thus, the additional emotion-based categories worsened the model predictions of aesthetic ratings for the walkers, suggesting that the aesthetic judgments are better explained by assuming that human walks form a single category.

### Objective and subjective prototypicality

After observing the strong correlation between prototypicality and aesthetic impressions, we next ask: what is the causal relationship between these variables? In general, correlational data afford multiple causal interpretations. However, in this case an additional constraint is apparent: model-derived prototypicality is an objective statistical measure that solely depends on the distribution of stimulus exemplars, whereas aesthetic impressions were measured by subjective human judgments. It seems logically impossible for a subjective measure to have a causal impact on an objective statistical measure on stimuli. A remaining question, however, is whether the causal path from objective prototypicality to aesthetic impression operates by a direct causal path, indirect path via some other variables (e.g., perceive naturalness of actions), or both.

While it is often possible in general that a third factor could cause both objective prototypicality and aesthetic experiences, it is paradoxical in this specific case: the third factor would need to influence the objective prototypicality, i.e., causing the estimate to be higher or lower, yet not itself be part of the objective prototypicality, i.e., not changing the estimate. For example, perhaps actors find it easier to perform neutral walks compared to happy, angry, and sad emotional walks, which led to differences in naturalness in the movements. However, this could not be considered as a third factor that could cause both the differences in objective prototypicality and aesthetic experiences, as the differences in naturalness manifest in the movements themselves and thus, would be part of the measure of objective prototypicality, rather than acting as a third factor that could influence the objective prototypicality.

We first examined the relationship between objective prototypicality derived from the single category model and subjective prototypicality (i.e., naturalness ratings) provided by observers from Experiment 3. Each video’s mean naturalness ratings and model-derived prototypicality are depicted in Fig. 2e. There was a clear positive correlation between the two measures, both before and after regressing out the emotion positivity ratings (with naturalness z-score: *r*(78) = 0.556, *p* < 0.001; with naturalness residuals after regressing out the emotion positivity: semipartial correlation *r*(78) = 0.540, *p* < 0.001), suggesting that typical movements were indeed associated with more natural impressions. This result was further supported by the similar patterns found with naturalness ratings (Fig. 2f) and modeled prototypicality (Fig. 3b) when we separated the results based on emotion categories: Neutral walkers were perceived as the most natural, followed by happy, sad, and angry walkers (*M*_happy_ = 3.5 (0.4), *M*_neutral_ = 4.0 (0.5), *M*_angry_ = 3.1 (0.5), *M*_sad_ = 3.5 (0.5); one-way ANOVA main effect with *z*-scores: *F*(3, 147) = 55.6, *p* < 0.001, *η*^*2*^_*p*_ = 0.531; all post hoc comparison with neutral walker: ts > 6.0, ps < 0.001 after Bonferroni correction). Moreover, the effect persisted after regressing out the emotion positivity ratings from the naturalness ratings (one-way ANOVA main effect with residuals: *F*(3, 147) = 58.4, *p* < 0.001, *η*^*2*^_*p*_ = 0.544; all post hoc comparison with neutral walkers: ts > 6.4, ps < 0.001 after Bonferroni correction). For emotion positivity ratings, we replicated the finding in Experiment 1 that happy walks were perceived as the most positive (*M*_happy_ = 3.9 (0.4), *M*_neutral_ = 3.5 (0.4), *M*_angry_ = 3.4 (0.5), *M*_sad_ = 2.5 (0.4); one-way ANOVA main effect with *z*-scores: *F*(3, 147) = 227, *p* < 0.001, *η*^*2*^_*p*_ = 0.823; all post hoc comparison with happy walkers: ts > 9.0, ps < 0.001 after Bonferroni correction).

Next, we asked if a movement’s model-derived prototypicality exerts its effect on aesthetic experience through a subjective impression of prototypicality. We conducted mediation analyses to examine both the direct effect (modeled prototypicality directly influenced aesthetic experience), and the indirect effect (subjective prototypicality mediated the effect on aesthetic experience). Because the same stimuli were used in Experiments 1 and 3, this analysis included both aesthetic judgments (from Experiment 1) and naturalness judgments (from Experiment 3). With a Sobel test and the method of permutation confidence interval (Taylor & MacKinnon, 2012), using data from both Experiments 1 and 3, we found a partial indirect causal relationship (Fig. 3c) both before and after regressing out the emotion positivity: Objective prototypicality influenced the subjective impression of the prototypicality of walks (i.e., naturalness ratings), which in turn influenced the aesthetic impression of the walks (z-score analyses: the Sobel test, *t*(77) = 4.84 *p* < 0.001; the permutation CI = [0.229, 0.538], which did not include zero; residual analyses: the Sobel test, *t*(77) = 5.18, *p* < 0.001; the permutation CI = [0.274, 0.604], which did not include zero). The gross direct relationship between the objective prototypicality and the aesthetic impression (z-score: slope = 0.566, *p* < 0.001; residuals: slope = 0.546, *p* < 0.001) was substantially weakened after removing the indirect effect of the subjective impression of prototypicality (z-score: slope = 0.183, *p* = 0.027; residuals: slope = 0.102, *p* = 0.115).

## Discussion

Using a combination of behavioral experiments and computational models, we made four main findings: (1) People share substantial consensus on how aesthetically pleasing a walk looks. (2) Human walks look more aesthetically pleasing when they expressed positive emotions; this preference depends on holistic processing and explicit recognition of human body configurations. (3) Aesthetic prototype effects can be observed in human actions: People find prototypical walks more aesthetically pleasing than atypical walks. (4) This effect was caused both directly by the prototypicality of the walk itself and indirectly through the mediation of the subjective impression of prototypicality.

The observed prototype effects indicate that human walks expressing different emotion states form a single category within a representational space, which opens a new dimension in the exploration of categorical processing. Beyond static objects and animals, dynamic events can form categories and afford the same kind of representational structures that lead to prototype effects. These categories likely form at the basic level (Rosch et al., 1976), as the emotional expressions and gender of the walkers does not divide the action representations into multiple categories (at least in the context of forming general impressions such as aesthetic impressions). This finding also suggested that categorical processing of walking is not involved in differentiating emotional states underlying walking actions and may mainly serve the function of action recognition. (Note that it is still possible that other aspects of action perception are sensitive to emotion and other social intentions underlying actions.)

At the same time, the demonstration of an aesthetic prototype effect in human walks also constitutes a unique approach to understanding human action aesthetics. In contrast to the focus on domain-specific explanations for aesthetic experiences based on dance movements (Cross et al., 2011) or sexualized features in human walks (Meskó et al., 2021; Morris et al., 2013), we showed that human actions are subject to the same domain-general aesthetic processes that operate over static categories (Ryali et al., 2020). This way, the general explanations of aesthetic preferences can also apply to human actions. As prototypical actions could reflect health and developmental stability in body movements (Møller & Swaddle, 1997), an aesthetic preference for prototypes may have general functional value (Chen et al., 2022a, 2022b; Halberstadt & Rhodes, 2003; Unkelbach et al., 2008; Vogel et al., 2021; Zajonc, 2001). Any particular general account remains speculative, and future work is needed to further examine the exact functions of these prototypical preferences.

These discoveries inform several practical fields. The discovered clear consensus of how good a walk looks suggests a new source of bias to navigate in our personal and professional lives. At the same time, the prototype effect introduces a design principle for depicting animated humans, developing virtual agents, and creating robots. The computational models used in this study can also be used for machine assistance in medical diagnosis, rehabilitations, and prosthetic limbs design (Pitkin, 2013), such as developing an early screening test for abnormal gaits, an evaluative scoring program for improvements from rehabilitation, or automatic design evaluations for how prosthetic limbs affect movements.

### Supplementary Information

**Additional file 1.** Posthoc tests for all main effects of emotion category across three experiments and the modeling results were reported here.

## Data Availability

Experiment 1’s design and analysis plan were preregistered and can be viewed here: https://aspredicted.org/KJD_33W. All materials, code, and data can be downloaded here: 10.17605/OSF.IO/87G3E. A demonstration of the experiments can be viewed online here: https://yi-chia-chen.github.io/walker-prototype-demo-expt/.

## Abbreviations

DTW: Dynamic time warping algorithm
px: Pixel

## Footnotes

1 The results of Experiment 2 here appeared to rely on how fast the points moved—the faster the points moved, the more emotionally positive and aesthetically pleasing the walker looked. We supported this observation by calculating the sum speed of the joints for each walker following these steps: (a) The x position of the head in each frame were subtracted from the x positions of all 15 joints. (b) Each joint’s travel distance in each frame was calculated based on the joint’s x and y positions. (c) The sum of distance traveled by all 15 joints throughout the video divided by the total duration of the video (5 s) gave us the sum speed of all joints. This speed measure was positively correlated with the group average of both emotion positivity and aesthetic z-scores (emotion positivity: *r*(78) = 0.704, *p* < 0.001; aesthetics: *r*(78) = 0.346, *p* = 0.002), as well as individual’s emotion positivity and aesthetic ratings (emotion positivity: *M* = 0.112, SD = 0.150, Range = [− 0.201, 0.365]), *t*(49) = 5.77, *p* < 0.001, Cohen’s *d* = 0.82; with 40/50 observers showing positive correlations, *p* < 0.001; aesthetics: *M* = 0.113*, *SD = 0.286, Range = [− 0.528, 0.675]), *t*(49) = 2.79, *p* = 0.007, Cohen’s* d* = 0.39; with 33/50 observers showing positive correlations, *p* = 0.015).

2 We focused on the emotion categories rather than the gender categories because a separate experiment with a new group of 50 subjects showed that the emotion categories were more discriminable than the gender categories (for emotion discrimination between four emotions: chance = 25%, *M*_accuracy_ = 48.4%, SD_accuracy_ = 7.7%, *t*(49) = 21.42, *p* < 0.001, Cohen’s *d* = 3.03; for gender discrimination between two gender: chance = 50%, *M*_accuracy_ = 59.9%, SD_accuracy_ = 6.3%, *t*(49) = 11.03, *p* < 0.001, Cohen’s *d* = 1.56). However, we still constructed two additional models considering the gender categories for additional information: the gender category model and the emotion-plus-gender category model. For the gender category model, we computed the average similarity only within the same gender (to the other 39 walks). For the emotion-plus-gender category model, we computed the average similarity only within the same expression and gender (to the other 9 walks). We again examined the relation between the model prototypicalities and the aesthetic ratings from Experiment 1. In both models, we again found that the more prototypical a walk was, the more aesthetically pleasing it appeared (for the gender category model, aesthetic z-scores average: *r*(78) = 0.564, *p* < 0.001, aesthetic residuals average: *r*(78) = 0.551, *p* < 0.001; for the emotion-plus-gender category model, aesthetic z-scores average: *r*(78) = 0.347, *p* = 0.002, aesthetic residuals average: *r*(78) = 0.423, *p* < 0.001). The gender category model’s performances did not differ significantly from the single category model (aesthetic *z*-scores average: *p* = 0.946; aesthetic residuals average: *p* = 0.866), while the gender-plus-emotion category model performed worse before regressing out the emotion positivity ratings (*p* = 0.007), and not significantly different after (*p* = 0.124).
