# Supplementary material for: Aesthetic preferences for prototypical movements in human actions
Source: Cogn Res Princ Implic. 2023 Aug 17;8:55. doi: 10.1186/s41235-023-00510-0 (PMC10435434; doi:10.1186/s41235-023-00510-0)
Supplement: Supplementary file 1 — Additional file 1. Posthoc tests for all main effects of emotion category across three experiments and the modeling results were reported here. [file 41235_2023_510_MOESM1_ESM.pdf]

Supplemental Materials

We conducted Bonferroni-corrected posthoc tests for all main effects of emotion category across three experiments and the modeling results, and report the corrected p-values below.

Experiment 1

Aesthetic Ratings

Emotion	Happy	Neutral	Angry	Sad
Happy		<.001	<.001	<.001
Neutral	<.001		<.001	<.001
Angry	<.001	<.001		>.999
Sad	<.001	<.001	>.999	

Emotion Positivity Ratings

Emotion	Happy	Neutral	Angry	Sad
Happy		<.001	<.001	<.001
Neutral	<.001		.016	<.001
Angry	<.001	.016		<.001
Sad	<.001	<.001	<.001	

Aesthetic Residuals

Emotion	Happy	Neutral	Angry	Sad
Happy		<.001	<.001	>.999
Neutral	<.001		<.001	<.001
Angry	<.001	<.001		<.001
Sad	>.999	<.001	<.001	

Experiment 2

Aesthetic Ratings

Emotion	Happy	Neutral	Angry	Sad
Happy		.012	>.999	<.001
Neutral	.012		.066	<.001
Angry	>.999	.066		<.001
Sad	<.001	<.001	<.001	

Emotion Positivity Ratings

Emotion	Happy	Neutral	Angry	Sad
Happy		<.001	>.999	<.001
Neutral	<.001		<.001	<.001
Angry	>.999	<.001		<.001
Sad	<.001	<.001	<.001	

Aesthetic Residuals

Emotion	Happy	Neutral	Angry	Sad
Happy		>.999	>.999	.001
Neutral	>.999		>.999	.010
Angry	>.999	>.999		.044
Sad	.001	.010	.044	

Experiment 3

Naturalness Ratings

Emotion	Happy	Neutral	Angry	Sad
Happy		<.001	<.001	>.999
Neutral	<.001		<.001	<.001
Angry	<.001	<.001		<.001
Sad	>.999	<.001	<.001	

Emotion Positivity Ratings

Emotion	Happy	Neutral	Angry	Sad
Happy		<.001	<.001	<.001
Neutral	<.001		.412	<.001
Angry	<.001	.412		<.001
Sad	<.001	<.001	<.001	

Naturalness Residuals

Emotion	Happy	Neutral	Angry	Sad
Happy		<.001	<.001	.089
Neutral	<.001		<.001	<.001
Angry	<.001	<.001		<.001
Sad	.089	<.001	<.001	

Single-category Model

Objective Prototypicalities

Emotion	Happy	Neutral	Angry	Sad
Happy		.024	.091	>.999
Neutral	.024		.002	.049
Angry	.091	.002		>.999
Sad	>.999	.049	>.999	
